# Impact of human gene annotations on RNA-seq differential expression analysis

**DOI:** 10.1186/s12864-021-08038-7

**Published:** 2021-10-08

**Authors:** Yu Hamaguchi, Chao Zeng, Michiaki Hamada

**Affiliations:** 1 Faculty of Science and Engineering, Waseda University, 55N-06-10, 3-4-1 Okubo Shinjuku-ku, Tokyo, 169-8555 Japan; 2 AIST-Waseda University Computational Bio Big-Data Open Innovation Laboratory (CBBD-OIL), 3-4-1, Okubo Shinjuku-ku, Tokyo, 169-8555 Japan; 3 Institute for Medical-oriented Structural Biology, Waseda University, 2-2, Wakamatsu-cho Shinjuku-ku, Tokyo, 162-8480 Japan; 4 Graduate School of Medicine, Nippon Medical School, 1-1-5, Sendagi, Bunkyo-ku, Tokyo, 113-8602 Japan

**Keywords:** RNA-seq, Differential expression analysis, Benchmarking, Gene annotation

## Abstract

**Background:**

Differential expression (DE) analysis of RNA-seq data typically depends on gene annotations. Different sets of gene annotations are available for the human genome and are continually updated–a process complicated with the development and application of high-throughput sequencing technologies. However, the impact of the complexity of gene annotations on DE analysis remains unclear.

**Results:**

Using “mappability”, a metric of the complexity of gene annotation, we compared three distinct human gene annotations, GENCODE, RefSeq, and NONCODE, and evaluated how mappability affected DE analysis. We found that mappability was significantly different among the human gene annotations. We also found that increasing mappability improved the performance of DE analysis, and the impact of mappability mainly evident in the quantification step and propagated downstream of DE analysis systematically.

**Conclusions:**

We assessed how the complexity of gene annotations affects DE analysis using mappability. Our findings indicate that the growth and complexity of gene annotations negatively impact the performance of DE analysis, suggesting that an approach that excludes unnecessary gene models from gene annotations improves the performance of DE analysis.

**Supplementary Information:**

The online version contains supplementary material available at (10.1186/s12864-021-08038-7).

## Background

Human gene annotations are still growing, with several being available for the human genome such as GENCODE [1] and RefSeq [2]. GENCODE is the default gene annotation for the Ensembl project and is focused on collecting nonsense transcripts, such as long non-coding RNAs (lncRNAs), pseudogenes, and alternative splicing. RefSeq is the oldest sequence database built by the National Center for Biotechnology Information (NCBI) and is widely used. These annotations are far from complete [3] and are continually updated. For example, in GENCODE human gene annotation release 31, released in 2019, a total of 17858 novel lncRNA transcripts, approximately 60% compared with the previous release, were added [1] (see Additional File 1: Figure S1). In addition, the growth of gene annotations has accelerated with the development and application of high-throughput sequencing technologies [4, 5]. Gene annotation provides information on gene models and is essential for differential expression analysis.

DE analysis is a primary application in RNA-seq analysis that can be applied to a diverse range of research subjects such as the identification of differences between tissues [6] and exploring biomarkers [7]. Generally, DE analysis consists of the following three steps: First, RNA-seq reads are mapped (aligned) to a reference genome or transcriptome. Second, the abundance of each gene or transcript is estimated from the alignments. Third, differentially expressed genes (DEGs) or transcripts are identified from abundance estimates for each sample using statistical methods. Gene annotation provides information on gene models required for splice-aware alignment and abundance estimation in DE analysis. With the increasing demand for RNA-seq, many tools for DE analysis have been developed [8, 9].

The impact of the complexity of gene annotations on DE analysis remains unclear. One of the difficulties faced during this analysis is the uncertainty of mapped reads, as RNA-seq reads are too short to uniquely map them to a gene locus or an isoform [10]. Complex gene models defined in gene annotation contribute to this uncertainty. Several benchmark studies have focused on analytical tools [11–21], whereas the impact of gene annotation is discounted. Although a few studies have focused on gene annotation [3, 22, 23], it is still unclear how the increasing complexity resulting from the growth of gene annotation affects DE analysis tools.

**Fig. 1:**
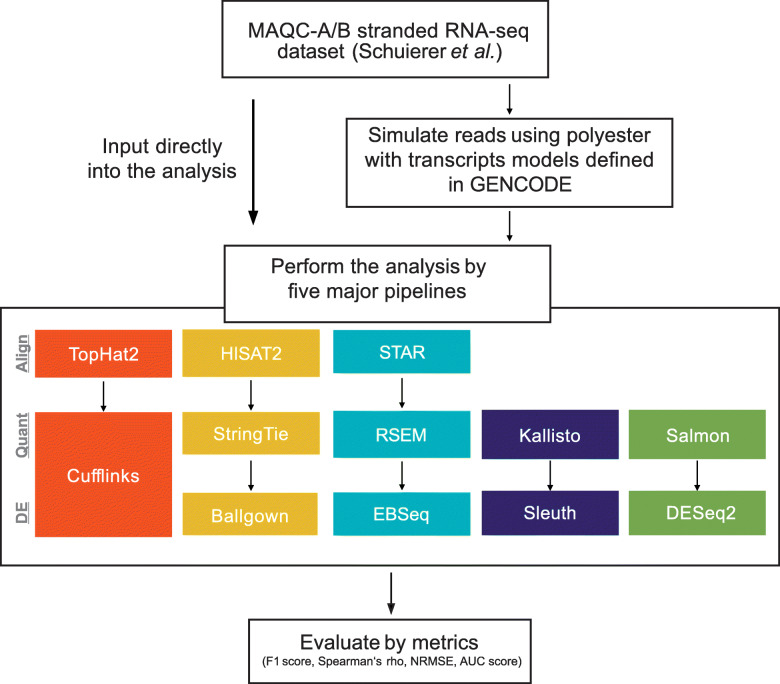
Overview of the evaluation. In this study, we evaluated the impact of the complexity of gene annotation using experimental and simulated datasets. Overview of evaluation After a literature survey, we selected five major DE analysis pipelines: TopHat2-Cufflinks, STAR-RSEM-EBSeq, HISAT-StringTie-Ballgown, Kallisto-Sleuth, and Salmon-DESeq2 (see also “Materials and methods”). We used a benchmarking RNA-seq dataset established by the MicroArray Quality Control (MAQC) project as both the experimental dataset and simulation input

Here, we assessed how the complexity of gene annotation affects DE analysis. First, we compared three human gene annotations, GENCODE, RefSeq, and NONCODE, and characterized these complexities using “mappability,” the fraction of reads derived from a transcript that aligned to the original transcript (see also “Materials and methods”). Next, we focused on GENCODE gene annotation and evaluated the impact of mappability on the performance of DE analysis using several metrics (a schematic illustration of the experimental design is shown in Fig. 1). Finally, we propose a filtering approach for gene models that uses mappability and abundance to improve DE analysis performance.

## Materials and methods

### Reference sequences and gene annotations

The GRCh38 reference genome (chromosomes only) and the GENCODE release 31 gene annotations (Comprehensive and Basic) were downloaded from the GENCODE website. RefSeq release 109 (20190607) gene annotations were downloaded from the NCBI website. RefSeq-Curated annotation was created by extracting “BestRefSeq” and “Curated Genomic” records from the full set of RefSeq. NONCODE version 5 was downloaded as a gene annotation of lncRNAs from the NONCODE website.

### Calculation of mappability

We utilized “mappability” as a metric to represent the complexity of gene annotation. Mappability is computed for each transcript or gene sequence, where a gene sequence is composed of one or multiple transcript sequences. Given a gene annotation, to calculate the mappability, we generated a set of subsequences (termed reads) from all transcript sequences (termed transcriptomes) using sliding windows of 50, 100 and 150 bases. These reads were then mapped to the transcriptome using Bowtie2 [24] with the ‘–sensitive’ option. When a read is mapped to N (N ≥1) distinct locations, we assign a 1/N read count for each mapped location. In the case that a transcript/gene contains a mapped location, a read count will be added to this transcript/gene. For a transcript/gene sequence *S*, suppose that *n* reads are generated from *S* and *m* reads are mapped (or assigned) to *S* (0<*m*≤*n*, where *m* can be a non-integer), then its mappability can be expressed as *m*/*n*. The value of mappability ranges from 0 to 1, with higher values indicating lower uncertainty for mapping reads to the corresponding transcript or gene; if the mappability is equal to 1 for a transcript, all the reads from the transcript are mapped to the original transcript). It should be noted that the above definition of mappability is slightly different from the original definition [25]. Mappability scores can evaluate the mapping complexity due to both intra- and intergenic shared sequences.

### Dataset

We used a benchmarking RNA-seq dataset established by the Microarray Quality Control (MAQC) project [26]. The dataset includes two types of samples: universal human reference from a mixture of tissue types (shown hereafter as MAQC-A) and human brain reference from brain tissue (shown hereafter as MAQC-B). In particular, we chose the stranded RNA-seq dataset generated by a third-party group [27] because the strand information was considered important to distinguish overlapping transcripts such as pairs of protein-coding and anti-sense RNAs. From the dataset, we extracted samples prepared by Ribo-zero, intact, and had sufficient input amount (> 5 ng) and used them for analysis. This dataset was used as input for the RNA-seq read simulation and the evaluation of real RNA-seq data. For comparison, MAQC-A samples were used as control for MAQC-B samples.

### Simulation of RNA-seq read datasets

We simulated an RNA-seq read dataset by the following steps: (1) Align MAQC-A/-B stranded RNA-seq reads to a reference genome using STAR [28], and estimate transcript abundance using RSEM [25] with custom parameters (described in Additional File 3); (2) Estimate parameters for each transcript *ϕ*_*i*_ and fold-change (fold-change was used as regulating factor *θ*_*i*_) of the negative binomial (NB) distribution with edgeR [29]; (3) Draw a read count for each transcript from the NB distribution (this read count was used as ground-truth); (4) Generate simulated RNA-seq read data using polyester read simulator [30] with the count matrix as input. Following a previous study [19], the count matrix of each group of samples is defined by the following formulas:

$$\begin{array}{*{20}l} & Y_{ij}^{Control} \sim NB(\mu_{i}, \mu_{i}(1 + \phi_{i}\mu_{i})), \\ & Y_{ij}^{Case} \sim NB(\theta_{i}\mu_{i}, \theta_{i}\mu_{i}(1 + \phi_{i}\theta_{i}\mu_{i})), \end{array} $$

where *Y*_*ij*_ is the read count of transcript isoform *i* in biological replicate *j*, *i*=1,…,*t* are transcript isoforms, *j*=1,…,*n* is biological replicates, *NB*(*mean,variance*) is a negative binomial distribution, *μ*_*i*_ denotes the mean value of isoform *i*, *μ*_*i*_(1+*ϕ*_*i*_*μ*_*i*_) denotes the variance of isoform *i*, *ϕ*_*i*_ is the dispersion parameter, and *θ*_*i*_ stands for the regulating factor of transcript isoform *i* between control and case samples. Note that *θ*_*i*_ was set to 1 for non-DE transcript.

As a result, simulated read data for a library size of 40 million reads, read length of 100 bases, and the layout of paired, replicate number n = 3 were obtained. The simulated read data were compared to the source experimental read data using countsimQC [31] (see Additional File 2).

### RNA-seq analysis pipelines

To choose tools for this evaluation, we surveyed the literature on current RNA-seq pipelines. Although DE analysis consists of several analysis steps, in this study, we focused on three major steps: read alignment, quantification, and DE testing. While choosing tools, we considered the following three important aspects: (1) availability to quantify at the transcript level; (2) algorithm comprehensiveness (alignment-based or alignment-free, and count-based or fragments per kilobase of transcript per million reads mapped (FPKM)-based); and (3) number of citations. As a result, we listed 12 tools from five pipelines (see Table 1). The parameters for each tool are described in Additional File 3. We defined genes or transcripts with |log2 fold-change| >= 1 and FDR < 0.05, as DE.

**Table 1:** Tools evaluated in this study

Tool	Abbrv. ^∗1^	Version	Category ^∗2^	#Citations ^∗3^	References	Year
TopHat2	Th	2.1.1	alignment	11740	[33, 34]	2009 (ver.1), 2013 (ver.2)
STAR	Sr	2.6.1d	alignment	5443	[28]	2013
HISAT	Hs	2.1.0	alignment	1799	[35, 36]	2015 (ver.1), 2019 (ver.2)
Cufflinks	Cu	2.2.1	assembly, quantification, DE	8102	[37, 38]	2010 (ver.1), 2013 (ver.2)
RSEM	Rs	1.3.1	quantification	4335	[25]	2011
StringTie	St	2.0.6	assembly, quantification	721	[39]	2015
Kallisto	Ka	0.46.1	quantification	312	[40]	2016
Salmon	Sa	1.5.0	quantification	517	[41]	2017
DESeq2	De	1.26.0	DE	6865	[42, 43]	2010 (ver.1), 2014 (ver.2)
EBSeq	Eb	1.26.0	DE	468	[44]	2013
Ballgown	Ba	2.18.0	DE	102	[45]	2015
Sleuth	Sl	0.30.0	DE	170	[46]	2017

^∗1^ Abbreviations specified above are used in this study.

^∗2^ The category of tools indicates the following: alignment, tools to map RNA-seq reads to reference, quantification, tools to estimate abundances, DE, and tools to identify DEs using the statistical method.

^∗3^ Number of citations reported by the Web of Science in October 2019

### Evaluation of mappability impact on simulated RNA-seq datasets

In quantification and DE evaluations, transcripts with under 0.25 CPM (approximately the same as 10 raw counts) in any of the samples of ground-truth were removed to avoid inflation of the metrics. All calculation results are saved in Additional File 4.

#### Alignment step

We evaluated the results of the alignment step with the following metrics: *Recall*=*TP*/(*TP*+*FN*),*Precision*=*TP*/(*TP*+*FP*),*F*1=2·(*recall*·*precision*)/(*recall*+*precision*), where True Positive (TP) is the number of reads mapped to the original transcript, False Positive (FP) the number of reads NOT mapped to the original transcript, and False Negative (FN) the number of unmapped reads.

#### Quantification step

The results of the quantification step were converted to a count matrix via tximport [32] (excluding Cuffdiff2). For Cuffdiff2, a count matrix was obtained from ‘isoforms.read_group_tracking’ file. Counts per million (CPM) were calculated for each transcript to express the corresponding abundance. For convenience, the CPM values are shown on the log2 scale hereafter. We evaluated the results of the quantification step with Spearman’s rho of log2 CPM and normalized root mean squared error (NRMSE) of log2 CPM between the estimated value and the ground-truth value.

#### DE step

We evaluated the results of the DE step with Spearman’s rho of log2 fold-change value, NRMSE of log2 fold-change value, and the Area Under the Receiver Operating Characteristic (ROC) Curve (AUC) between the estimated value and the ground-truth value. We defined transcripts with a *θ* greater than or equal to 2 in absolute values as true DEs. True positives (TP), false positives (FP), true negatives (TN), and false negatives (FN) are defined based on a comparison between the estimated differentially expressed call and true DEs.

### Evaluation of mappability impact on experimental RNA-seq datasets

We downloaded the TaqMan Quantitative Reverse Transcription Polymerase Chain Reaction (qRT–PCR) measurements provided by the MAQC project from the Gene Expression Omnibus (GEO) under accession number GSE5350, and used as a “gold-standard”. We converted the RefSeq gene ID to GENCODE gene ID using the conversion metadata provided by GENCODE. Following a previous study [25], non-expressed genes were filtered. As a result of conversion and filtering, 839 genes expressed in both MAQC-A and MAQC-B were obtained.

We evaluated the experimental RNA-seq dataset with Spearman’s rho of log2 fold-change, and NRMSE of log2 fold-change between the RNA-seq estimated value and the TaqMan qRT–PCR measurements at the gene-level. The Kallisto-Sleuth pipeline was excluded from this evaluation because it cannot output the gene-level fold-change value. Furthermore, genes with a mappability of 1 were excluded to avoid being occupied by a single value. Finally, we evaluated 502 genes.

To confirm the tendency of false positives in these pipelines, we also counted the number of DEs detected by regular comparison (MAQC-A vs. MAQC-B) and mock comparison (MAQC-A vs. MAQC-A) for all transcripts defined in the annotation at the transcript-level.

## Results

### Gene model complexity was significantly different among human gene annotations

First, to clarify the differences among human gene annotations, we summarized basic statistics (see Table 2). For this analysis, we used three gene annotations: GENCODE, RefSeq, and NONCODE. To confirm the difference in transcript selection within an annotation, GENCODE and RefSeq were compared with their subsets, GENCODE-Basic and RefSeq-Curated, respectively (see “Materials and methods” for details of these annotations). NONCODE is a gene annotation that consists of only lncRNAs. NONCODE was added to this analysis to confirm the differences in RNA type. Most of the transcripts defined in RefSeq were aggregated in the same gene locus, and it was difficult to identify the original transcripts of RNA-seq reads. Compared with GENCODE, RefSeq showed a decreased average percentage of unique exons per gene (70.4% for RefSeq vs. 85.5% for GENCODE), a lower genomic coverage of exon regions (4.11% vs. 4.72%), and a higher average number of transcripts per gene (4.09 vs. 3.74). In GENCODE-Basic, the uncertainty of mapping reads to the annotated transcriptome was lower than that of GENCODE. Compared with GENCODE, GENCODE-Basic showed an increased average percentage of unique exons per gene (89.0% for GENCODE-Basic vs. 85.5% for GENCODE) and a decreased average number of transcripts per gene (1.79 vs. 3.74). Note that, in GENCODE-Basic, the comprehensiveness of isoforms was also reduced. In RefSeq-Curated, the uncertainty for mapping reads was reduced compared to RefSeq. It should be noted that the comprehensiveness of genes, isoforms, and RNA types was reduced. Compared with RefSeq, RefSeq-Curated showed an increased average percentage of unique exons per gene (75.1% for RefSeq-Curated vs. 70.4% for RefSeq) and significant decreases in the number of genes (28784 vs. 39280) and transcripts (73442 vs. 160796). This result was caused by the exclusion of most non-coding RNAs (ncRNAs) by the manual curation process of RefSeq. NONCODE consists of gene loci that have a simpler gene model than other gene annotations. Compared to GENCODE and RefSeq, NONCODE showed the highest average percentage of unique exons per gene (95.7% for NONCODE vs. 85.5% for GENCODE vs. 70.4% for RefSeq) and the lowest average number of transcripts per gene (1.79 vs. 3.74 vs. 4.09%), although it showed a similar level of genomic coverage of exon regions to GENCODE (4.71% for NONCODE vs. 4.72% for GENCODE).

**Table 2:** Basic statistics of major human gene annotations

	GENCODE	GENCODE-Basic	RefSeq	RefSeq-Curated	NONCODE
Release	31	31	109.20190607	109.20190607	5
# of genes	60603	60603	39280	28784	96308
# of transcripts	226882	108243	160796	73442	172216
Genomic coverage of exon regions ^∗1^	4.72%	3.88%	4.11%	2.81%	4.71%
Avg. # of transcripts per gene	3.74	1.79	4.09	2.55	1.79
Avg. percentage of unique exons per gene ^∗2^	85.5%	89.0%	70.4%	75.1%	95.7%

^∗1^ Non-coding gene loci included.

^∗2^ Average percentage of exons with distinct junctions for each gene.

**Fig. 2:**
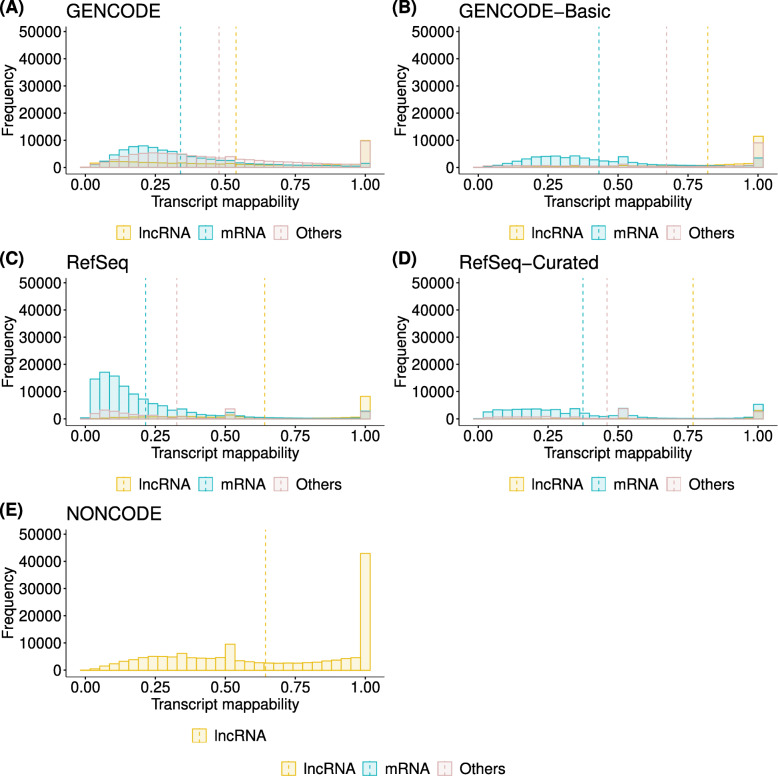
Complexity of gene models was significantly different among human gene annotations. We calculated mappability, the fraction of reads derived from a transcript that aligned to the original transcript, using 100 bases length reads for each transcript annotated in major human gene annotations (see also “Materials and methods”). The value of mappability ranges from 0 to 1, with higher values indicating lower uncertainty for mapping reads. (A)–(E) show the distribution of transcript mappability for GENCODE, GENCODE-Basic (a subset of GENCODE), RefSeq, RefSeq-Curated (a subset of RefSeq), and NONCODE, respectively. Colored bars indicate the frequency of mRNAs (blue), lncRNAs (yellow), and other biotypes (red). Dotted vertical lines indicate the average mappability for each gene annotation

Next, to quantify the complexity of gene models in more detail, we calculated the transcript mappability, the fraction of reads aligned to its original transcript. NONCODE showed the highest average mappability, followed by GENCODE-Basic, GENCODE, RefSeq-Curated, and RefSeq. Unlike other annotations in RefSeq, distribution peaks were observed in the range of low mappability (0.069–0.10) (see Fig. 2C). These transcripts with low mappability were mainly generated by automated annotation because they have been excluded from RefSeq-Curated (see Fig. 2D). Compared with GENCODE, GENCODE-Basic showed higher average mappability (0.58 for GENCODE-Basic vs. 0.44 for GENCODE; see Fig. 2A and B). This change was caused by the drastic exclusion of ncRNAs, including non-stop decay, retained intron, nonsense-mediated decay, and lncRNA. In NONCODE, most transcripts showed high mappability (see Fig. 2E). This result indicates that most transcripts defined in NONCODE are uniquely mappable to the NONCODE transcriptome. In each annotation, protein-coding genes showed lower mappability than lncRNAs, and their gene models tended to be complex. As expected, In GENCODE and NONCODE, the mean mappability improved with increasing read length (between 50 and 150 bases, approximately 0.043) (see Additional File 1: Figure S2, Fig. 2 and Additional File 1: Figure S3). However, in RefSeq, the mappability improvement with increasing read length was smaller (0.011) than GENCODE and NONCODE because most RefSeq transcripts consisted of shared exons.

These results show that complexity is significantly different among human gene annotations owing to differences in data sources and collected RNA types. Accordingly, the choice of gene annotation results in differences in DE analysis outcomes.

### Increasing mappability improves the performance of DE analysis

To clarify the impact of mappability on DE analysis, we divided the transcripts defined in GENCODE gene annotation into three equal-sized groups according to transcript mappability and evaluated these groups. Because the abundance of transcripts affected the quantification accuracy [47], we compared metrics within a group of transcripts with similar expression levels. To avoid bias resulting from specific tools and algorithms, we chose five RNA-seq pipelines, including STAR-RSEM-EBSeq, HISAT-StringTie-Ballgown, Kallisto-Sleuth, Tophat2-Cufflinks, and Salmon-DESeq2 (see “Materials and methods” and Table 1).

**Fig. 3:**
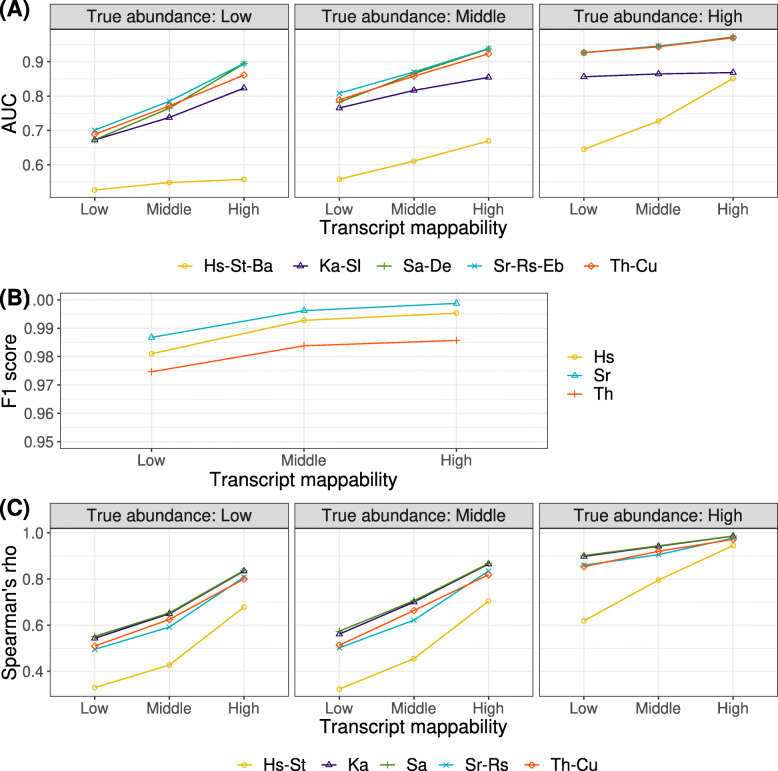
Impact of transcript mappability on the performance of DE analysis with a simulated dataset. We divided transcripts into three equal-sized groups of low, middle, and high values of transcript mappability and true transcript abundance. Intervals of transcript mappability were as follows: low, [0.0107, 0.257); middle, [0.257, 0.472); and high,[0.472, 1]. Intervals of mean true transcript abundance (CPM) for DE step evaluation were as follows: low, [0.539, 2.65); middle, [2.65, 9.68); and high, [9.68, 5.54 ×10^4^]. Intervals of true transcript abundance (CPM) for quantification step evaluation were as follows: low, [0.250, 1.10); middle, [1.1, 5.01); and high, [5.01, 6.91×10^4^]. (A) Relationship between the AUC score and transcript mappability faceted by mean true transcript abundance. (B) Relationship between F1 score and transcript mappability during the alignment step. (C) Relationship between Spearman’s rho of CPM value and transcript mappability faceted by true transcript abundance. Metrics were calculated for all RNA types. Hs, HISAT; St, StringTie; Ba, Ballgown, Ka; Kallisto, Sl; Sleuth, Sa; Salmon, De; DESeq2, Sr; STAR, Rs; RSEM, EB; EBSeq, Th; TopHat2, Cu; Cufflinks (also see Table 1)

First, we evaluated the impact of mappability on DE analysis with the simulated dataset. AUC scores improved monotonically with increasing transcript mappability, excluding HISAT-StringTie-Ballgown (see Fig. 3A). The improvement was particularly significant (with a range of 0.15–0.22) in the low transcript abundance group. For the HISAT-StringTie-Ballgown pipeline, mappability did not significantly affect the AUC score in the low true transcript abundance group. However, in the high true transcript abundance group, a significant improvement was observed (0.21). The default filtering criteria of Ballgown excluded values with small variances. This filtering resulted in only a small set including 754–1541 transcripts that were evaluated as the group with low transcript abundance. Thus, the AUC score for this group was not reliable. Increasing mappability and true transcript abundance improved the performance of DE analysis.

Next, to identify how mappability affects the DE analysis pipeline, we evaluated each step of the DE analysis, including alignment and quantification, in the simulated dataset. In the alignment step evaluation, F1 scores improved slightly with increasing transcript mappability (see Fig.3B). Each tool showed high performance (> 0.97) and equivalent sensitivity to mappability. In the quantification step evaluation, the Spearman’s rho of log2 CPM improved monotonically with increasing transcript mappability (see Fig. 3C). The improvement was particularly significant (ranging from 0.29–0.35) in the low transcript abundance group. Algorithms that correct uncertainty in mapping reads, such as the expectation maximization (EM) algorithm [48], did not work as expected in transcripts with low expression levels. Furthermore, misassigned reads to low-abundance transcripts from high-abundance transcripts sharing partial sequences may cause large errors in the estimates of low-abundance transcripts. This tendency of the quantification step is consistent with that of the DE step.

One idea to improve performance is excluding non-expressed transcripts from gene annotations to reduce complexity. To explain this idea, we created a tailored GENCODE gene annotation and evaluated the performance of DE analysis with that annotation (Additional File 1: Figure S4 and Additional File 4). As expected, the performance of the DE analysis improved. AUC scores slightly increased by an average of 0.013 in all pipelines tested.

**Fig. 4:**
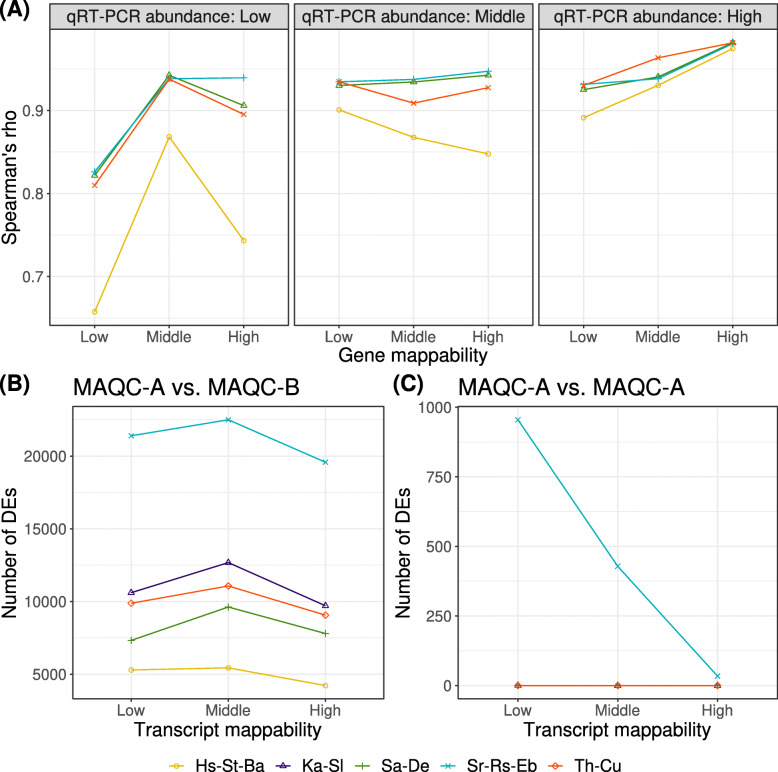
Impact of mappability on the performance of DE analysis with an experimental dataset. We divided the transcripts into three equal-sized groups (low, middle, and high) according to the values of gene- and transcript- mappability and qRT–PCR measurement. Intervals of gene mappability were as follows: low, [0.46, 0.961); middle, [0.961, 0.991); and High, [0.991, 1). Genes with a mappability of ‘1’ were excluded from this grouping to avoid one group being occupied by one value. Intervals of qRT-PCR measurements (mean relative expression for internal control gene) were as follows: low, [0.000512, 0.0621]; middle, [0.0621, 0.353); and High, [0.353, 38.8]. Intervals of transcript mappability were as follows: low, [0.001, 0.255); middle, [0.255, 0.508); and High, [0.508, 1.00]. (A) Relationship between Spearman’s rho and gene mappability determined by qRT–PCR. Metrics were calculated for qRT–PCR validated 502 genes (see “Materials and methods for details of list of these genes). The Kallisto-Sleuth pipeline was excluded from this evaluation because it cannot output the gene-level fold-change value. (B) and (C) show the number of DEs compared with regular and mock comparisons, respectively. Metrics were calculated for all transcripts as defined by GENCODE. Hs-St-Ba; HISAT-StringTie-Ballgown, Ka-Sl; Kallisto-Sleuth, Sa-De; Salmon-DESeq2, Sr-Rs-EB; STAR-RSEM-EBSeq, Th-Cu; Tophat2-Cufflinks

Finally, we validated these results with the experimental dataset because the simulation may lack some RNA-seq dataset characteristics. The following restrictions were noted when using the experimental dataset: (1) qRT–PCR data as ground-truth were limited in size (only 1044 probes) and were measured at the gene level; (2) it is biased toward those with high mappability; (3) true DE cannot be defined. Based on mappability, we divided genes and transcripts defined in the GENCODE gene annotation into three equal-sized groups. We used two metrics, including Spearman’s rho of fold-change against qPCR measurements and the number of DEs. Spearman’s rho of fold-change tended to be lower in the low gene mappability group than in the middle and high mappability groups (see Fig. 4A). Note that few observations (20–40) passed the DE step filtering in the low qPCR abundance and high gene mappability group, which had more missing values than other groups. We compared the number of DEs between regular comparisons (MAQC-A vs. MAQC-B) and mock comparisons (MAQC-A vs. MAQC-A) (see Fig. 4B and C). Regular comparisons showed a consistent number of DEs for all tools (a range of 4175–22535) independent of mappability. However, mock comparisons showed that only zero or one DE was detected, except for the STAR-RSEM-EBSeq pipeline. For the STAR-RSEM-EBSeq, particularly in the low mappability group, many DEs were detected (796–1118). In particular, EBSeq seemed more sensitive to mappability than other tools because it considers the uncertainty of mapping reads [44]. We conclude that increasing mappability tends to improve DE analysis performance with the experimental dataset, which is consistent with that of the simulated dataset.

These results show that increasing mappability improves the performance of DE analysis. Furthermore, the impact of mappability occurs mainly in the quantification step and systematically propagates downstream of the DE analysis.

## Discussion

We assessed here how the complexity of gene annotation affects DE analysis using mappability. We show that complexity was significantly different among human gene annotations. We also show that increasing mappability improved the performance of the DE analysis.

Our results show that the increasing complexity of gene annotation adversely affected DE analysis. Wu et al. [23] evaluated the impact of human gene annotation choice on RNA-seq expression estimates. They defined the complexities of gene annotations in terms of the relative rank of the number of genes, isoforms, and exons and demonstrated that more complex annotation results in a smaller correlation between RNA-seq fold-change and qRT–PCR fold-change. Our results are consistent with these findings. For studies that emphasize accuracy and clarity, less complex gene annotations such as GENCODE-Basic or RefSeq-Curated may be preferred. Note that our results are based on an evaluation that ignores unannotated transcripts. Zheng et al. [49] reported that using partial (RNA type-specific) gene annotation such as NONCODE results in overestimated expression compared to a more comprehensive annotation. Varabyou et al. [50] suggest that an assembly-based method such as StringTie is more robust against transcriptional noise than annotation-based methods such as Salmon and Kallisto. Assignment of noise-derived RNA-seq reads to noise-derived gene models reduces overestimation. Note that gene models constructed from small datasets are unreliable and difficult to interpret. In summary, both the comprehensiveness and complexity of gene annotation are important for experimental DE analysis.

We propose excluding unnecessary gene models from gene annotation to improve the performance of DE analysis. Chen et al. [3] suggest that the integration of multiple gene annotations improves the comprehensiveness and sensitivity of DE analysis. Our results suggest that careless gene annotation integration is not recommended because of increasing complexity. However, the combination of integration and filtering of gene models considering redundancy may improve the performance of DE analysis. Our results, using a tailored gene annotation, support this idea. It is not easy to know non-expressed transcripts using experimental datasets. One approach to this problem is to filter out low abundance and low mappability transcripts to obtain clear results. Our results show that the estimation of transcripts with low abundance and mappability was unreliable. Filtering based on abundance has been used to reduce the number of tests in the DE step, introducing a mappability representing uncertainty for mapping reads and leading to a better exclusion of noisy estimates. Another idea is to consider the sequencing conditions. A typical RNA-seq library does not contain non-poly-A or small RNAs. Because gene models corresponding to these RNAs that cannot be captured become analytical noise, excluding them may improve performance. However, it is difficult to obtain information on the presence of poly-A in each transcript.

In future work, we will evaluate non-annotation-based methods such as [51, 52]. We will also examine the extent to which annotations fit the experimental RNA-seq datasets. Developing a method for integrating and tailoring gene annotations would also be useful.

## Conclusions

In this study, we assessed how the complexity of gene annotation affects DE analysis using mappability. We observed that the complexity was significantly different among the three human gene annotations, including GENCODE, RefSeq, and NONCODE, and show that the choice of gene annotation is important in DE analysis. We also observed that increasing mappability improved the performance of DE analysis. Our findings indicate that the growth and complexity of gene annotation negatively affects the performance of DE analysis. We propose an approach that excludes unnecessary gene models from gene annotation using mappability and abundance to improve the performance of DE analysis.

## Supplementary Information

**Additional file 1** Supplementary figures.

**Additional file 2** Comparison of characteristic features across the count dataset (HTML).

**Additional file 3** Parameters used for each tool.

**Additional file 4** All metrics (Microsoft Excel).

## Data Availability

All scripts used in this study are available on the github.com repository (https://github.com/hmdlab/eval_rnaseqde_map). The datasets simulated during this study can be generated by the above scripts. GENOCDE gene annotation files are available on the GENCODE website (https://www.gencodegenes.org/). RefSeq gene annotation file is available on the RefSeq website (https://www.ncbi.nlm.nih.gov/refseq/). NONCODE gene annotation file is available on the NONCODE website (http://www.noncode.org/). The MAQC RNA-seq dataset is available on the ENA website (https://www.ebi.ac.uk/ena/browser/home) under accession number SRP097611.
