# Supplementary material for: Impact of human gene annotations on RNA-seq differential expression analysis
Source: BMC Genomics. 2021 Oct 8;22:730. doi: 10.1186/s12864-021-08038-7 (PMC8501603; doi:10.1186/s12864-021-08038-7)
Supplement: Supplementary file 1 — Additional file 1 Supplementary figures. [file 12864_2021_8038_MOESM1_ESM.pdf]

Additional file 1: Supplementary figures

Supplementary figures

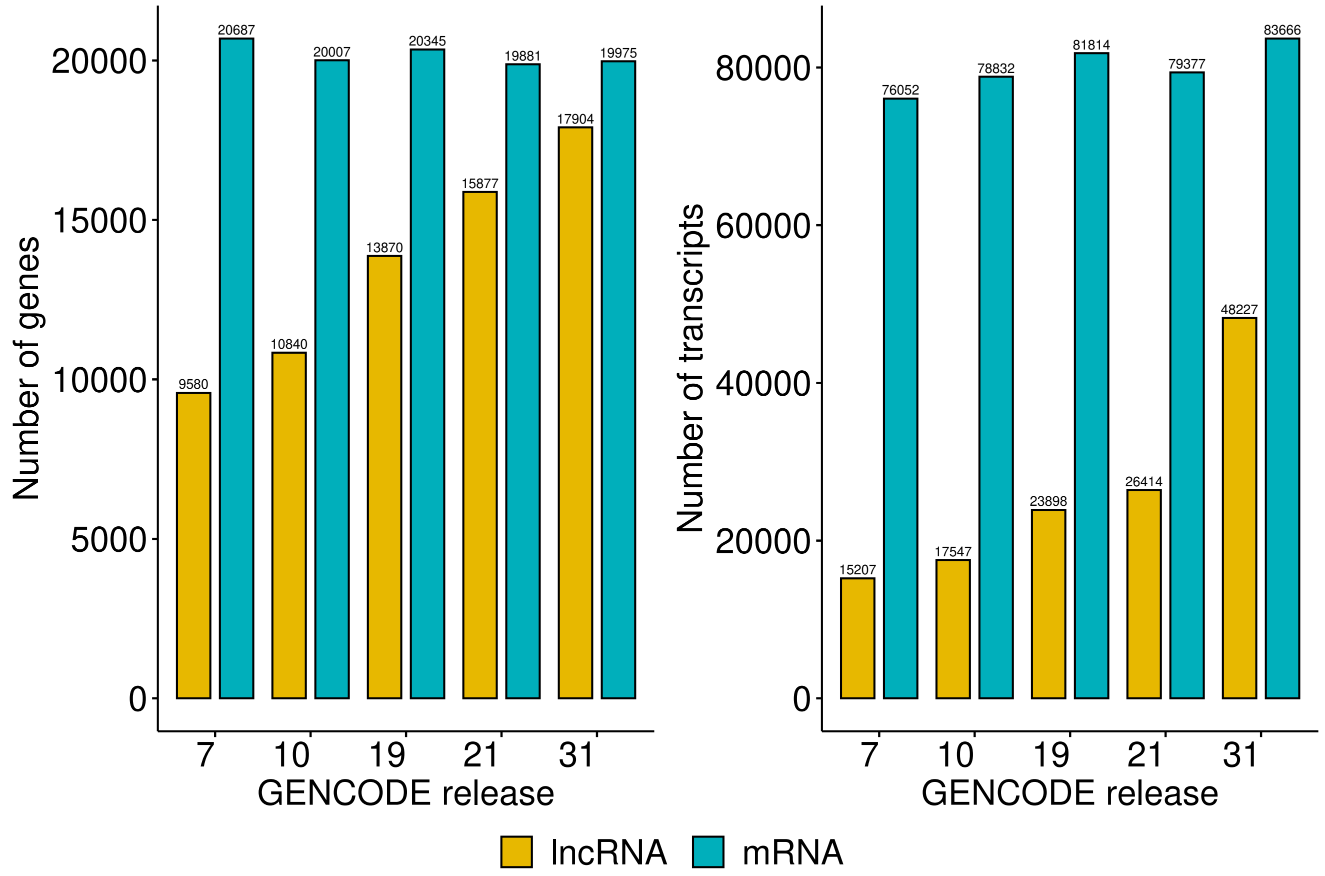

Figure S1. Growth of GENCODE gene annotation. The left and right panels show the number of gene loci and transcripts annotated in GENOCDE gene annotation, respectively. Only the past major releases and the latest release 31 at the time of writing are included. Colors indicate RNA types.

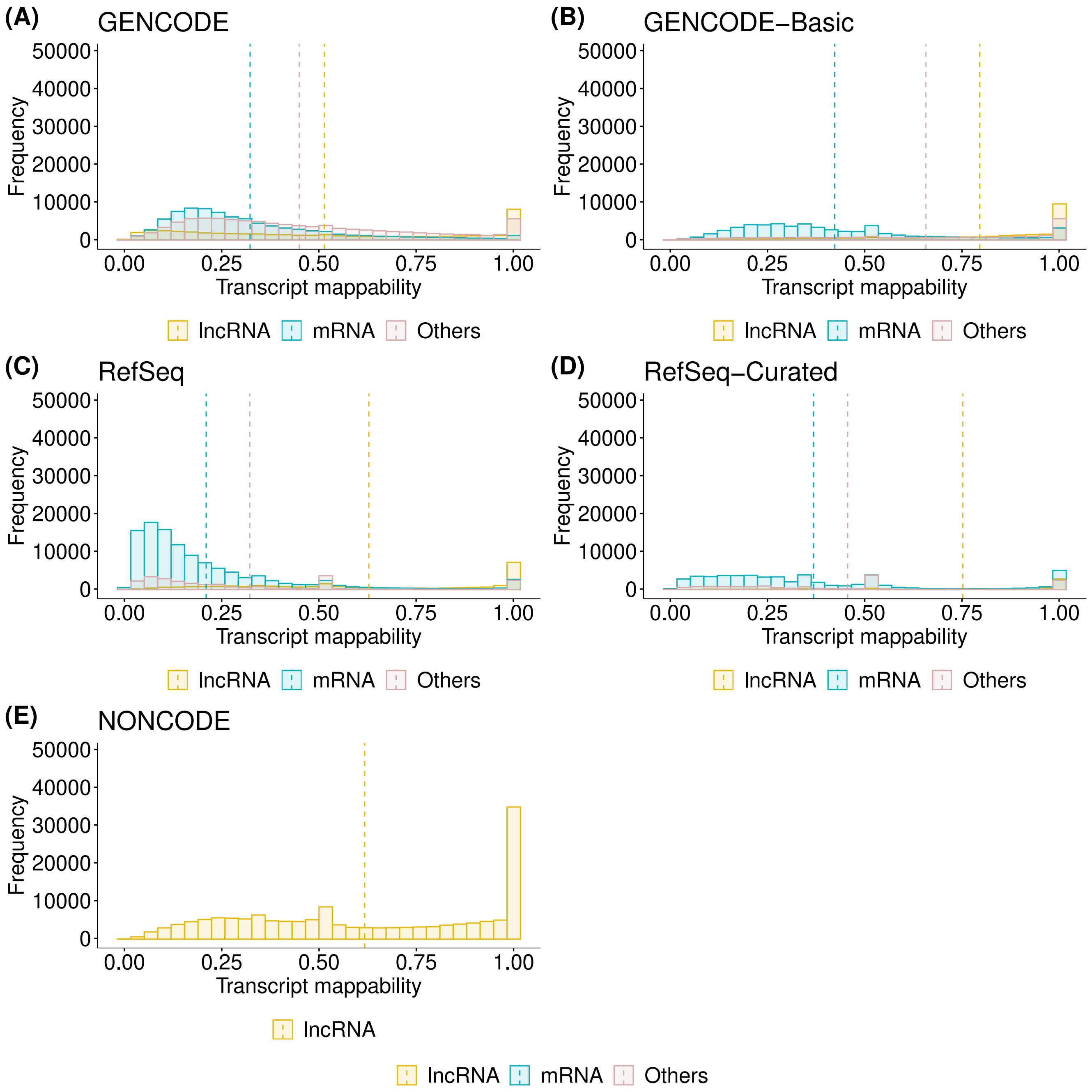

Figure S2. Complexity of gene models was significantly different among human gene annotations. We calculated mappability using 50 bases length reads. (A)–(E) show the distribution of transcript mappability for GENCODE, GENCODE-Basic (a subset of GENCODE), RefSeq, RefSeq-Curated (a subset of RefSeq), and NONCODE, respectively. Colored bars indicate the frequency of mRNAs (blue), lncRNAs (yellow), and other biotypes (red). Dotted vertical lines indicate the average mappability for each gene annotation.

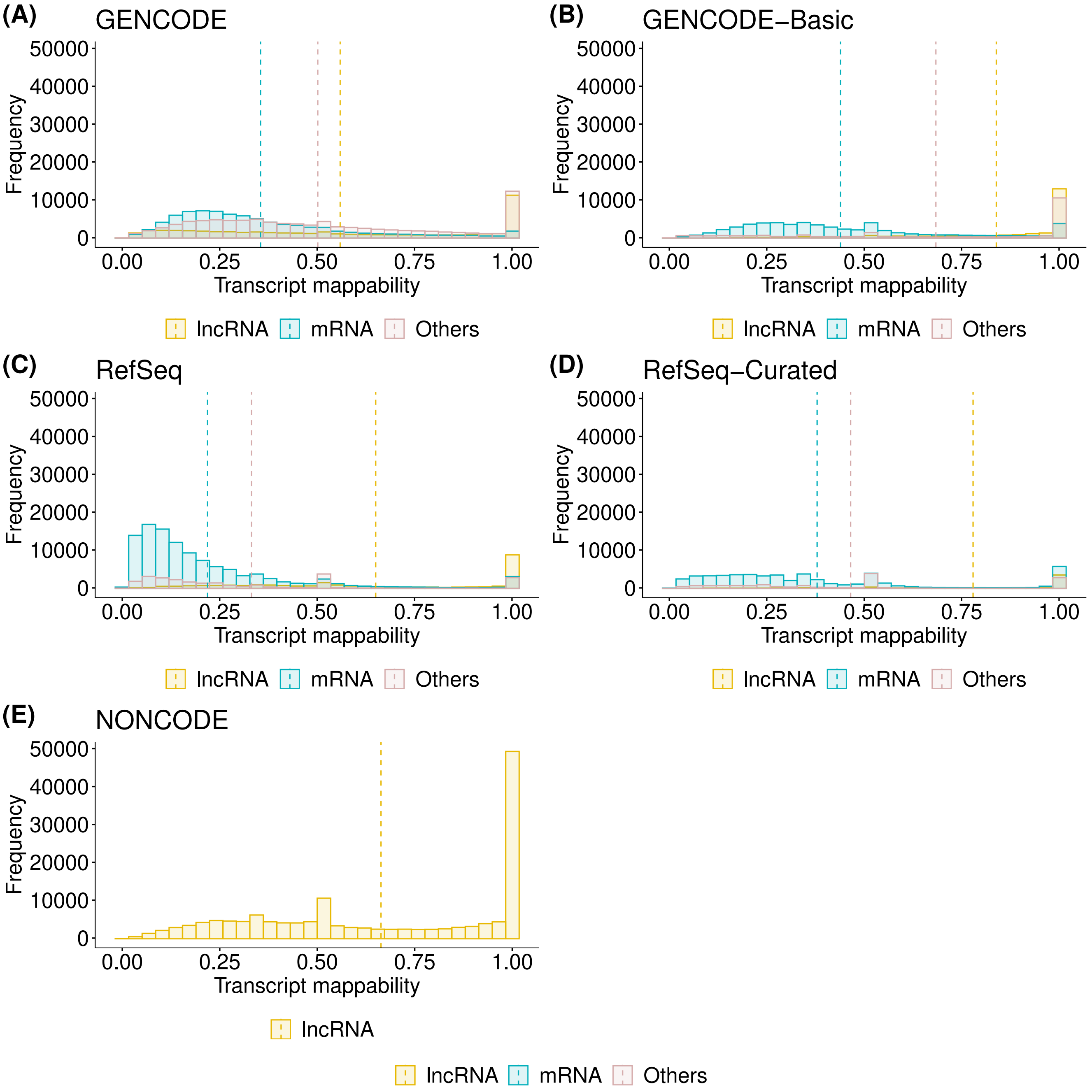

Figure S3. Complexity of gene models was significantly different among human gene annotations. We calculated mappability using 150 bases length reads. (A)–(E) show the distribution of transcript mappability for GENCODE, GENCODE-Basic (a subset of GENCODE), RefSeq, RefSeq-Curated (a subset of RefSeq), and NONCODE, respectively. Colored bars indicate the frequency of mRNAs (blue), lncRNAs (yellow), and other biotypes (red). Dotted vertical lines indicate the average mappability for each gene annotation.

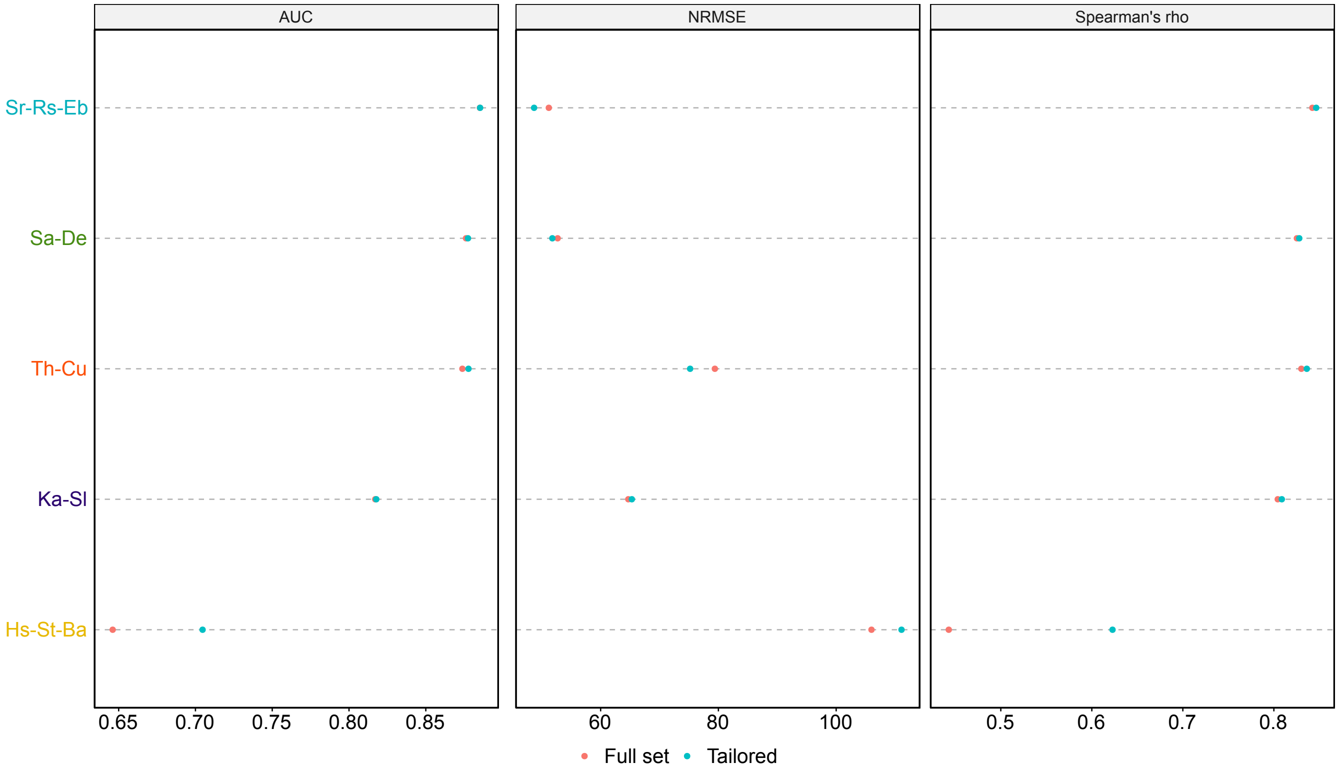

Figure S4. Comparison of metrics with full set and tailored annotation on DE step. Three metrics evaluated analysis results with full set gene annotations and tailored gene annotations: AUC, RMSE, and Spearman's rho. These metrics were calculated for all transcripts expressed. Colors indicate the annotation used. Abbreviations indicate combinations of the following tools: Hs-St-Ba; HISAT-StringTie-Ballgown, Sa-De; Salmon-DESeq2, Ka-Sl; Kallisto-Sleuth, Th-Cu; Tophat2-Cufflinks.
