# Supplementary material for: Impact of human gene annotations on RNA-seq differential expression analysis
Source: BMC Genomics. 2021 Oct 8;22:730. doi: 10.1186/s12864-021-08038-7 (PMC8501603; doi:10.1186/s12864-021-08038-7)
Supplement: Supplementary file 3 — Additional file 3 Parameters used for each tool. [file 12864_2021_8038_MOESM3_ESM.pdf]

Additional file 3: The parameters for each tool

1 polyester

1.1 simulate_experiment_countmat():

```
paired = TRUE,
readlen = 100,
error_rate = 0.005,
bias = "none",
strand_specific = TRUE,
seed = 12345
```

2 TopHat2-Cufflinks pipeline

2.1 TopHat2

```
–num-threads 4
```

2.2 Cuffdiff2

```
–emit-count-tables
–max-bundle-frags 100000000
–num-threads 6
```

3 HISAT2-StringTie-Ballgown pipeline

3.1 HISAT2

```
–seed 12345
–new-summary true
–no-mixed true
–threads 2
```

3.2 StringTie

```
-e
-B
```

4 STAR-RSEM-EBSeq pipeline

4.1 STAR

```
–readFilesCommand zcat
```

```
–readNameSeparator "’|’"
–limitBAMsortRAM 48000000000
–outReadsUnmapped Fastx
–outSAMtype "BAM SortedByCoordinate"
–quantMode TranscriptomeSAM
–outSAMattributes All
–outSAMstrandField intronMotif
–outSAMheaderHD "’@HD VN1.4 SOcoordinate’"
–runThreadN 2
–runRNGseed 777
```

4.2 RSEM

```
–bam
–estimate-rspd
–calc-ci
–seed 12345
–no-bam-output
–num-threads 4
–ci-memory 61440
```

5 Kallisto-Seleuth

5.1 Kallisto

```
–seed 12345
-b 100
–pseudobam
–genomebam
```

5.2 Sleuth

5.2.1 sleuth_results():

```
test_type = "wt",
show_all = FALSE,
pval_aggregate = FALSE
```

6 Salmon-DESeq2 pipeline

6.1 Salmon

Execute with default parameters.

6.2 DESeq2

6.2.1 nbinomWaldTest():

Execute with default parameters.
